# We're closer than ever to ending AIDS—let's not lose ground

**DOI:** 10.3389/fpubh.2025.1655240

**Published:** 2025-09-26

**Authors:** Peter Sands

**Affiliations:** Executive Director of the Global Fund to Fight AIDS, Tuberculosis and Malaria, Geneva, Switzerland

**Keywords:** HIV infection, global health, health policy, antiretroviral therapy, international cooperation

## Introduction

HIV was once a seemingly insurmountable global crisis. Yet over the past two decades, we have made extraordinary progress in prevention, treatment, and care. Through global cooperation – anchored in national leadership, community engagement, and investment from initiatives like the Global Fund and PEPFAR – tens of millions of lives have been saved.

But today, this hard-won progress is at risk. The reduction in funding for global health programs reflects a troubling erosion of political will. At a time when ending HIV as a public health threat is within reach, we risk faltering just short of the finish line.

This is a pivotal moment. The question is no longer whether we can defeat HIV and AIDS – but whether we will maintain the resolve to do so.

## The triumph of collective action

Since its founding in 2002, catalyzed by the G8 Summit in Genoa, the Global Fund has helped demonstrate what collective global action can achieve. At the height of the epidemic, AIDS was claiming nearly 2 million lives every year. Today, in countries such as Botswana, Eswatini, Kenya, Malawi, Rwanda, Zambia and Zimbabwe, the 95-95-95 targets have been reached: 95% of people living with HIV know their status, 95% of those people are on treatment, and 95% of those on treatment are virally suppressed (1).

These successes are not accidents. They are the result of decades of sustained effort by governments, affected communities, civil society, faith-based organizations and the private sector. Together, we have shown that multilateralism and solidarity are not abstract ideals – they are powerful, lifesaving strategies.

But we must not take them for granted.

## Why continued commitment matters

Despite these remarkable gains, HIV and AIDS are far from over. For the first time in the epidemic's history, more new infections are now occurring outside of sub-Saharan Africa than within it. While, between 2010 and 2024, the number of new HIV infections decreased by 56% in sub-Saharan Africa, this number has increased by 94% in the Middle East and North Africa, 13% in Latin America, and 7% in Eastern Europe and Central Asia (2). This rising incidence is often driven by stigma, criminalization, and weak health systems (1). Furthermore, structural barriers such as socioeconomic inequalities and gender disparities remain a major cause of uneven progress not only among but also within countries and disproportionate risk for vulnerable populations. Now is not the time for complacency. The global HIV response is only as strong as its weakest health program – and those programs are under increasing strain.

New tools, such as long-acting HIV prevention options like lenacapavir for pre-exposure prophylaxis (PrEP), offer unprecedented potential to curb transmission. Combined with antiretroviral therapy (ART), which enables people living with HIV to lead long, healthy lives, we are closer than ever to defeating this disease.

But these gains depend on sustained investment. ART is a lifelong commitment, and its success hinges on continuous support. HIV-related comorbidities – such as tuberculosis and certain non-communicable diseases – further stretch already fragile health systems. Slashing funding now would be a false economy, jeopardizing not just health outcomes, but also decades of progress in health infrastructure.

## The Global Fund's essential role

At the heart of this effort stands the Global Fund – data-driven, efficient and focused on equity. Through innovative financing mechanisms and partnership models, we have helped drive down the cost of ART from US$10,000 in 2002 to as little as US$37 per person per year today, while expanding access to the latest cutting-edge prevention tools.

In severely resource-constrained environments, ensuring rapid, affordable, and equitable access to biomedical and other technological innovations is imperative. With that aim in mind, the Global Fund has increased its engagement with innovators and technical experts to identify promising pipeline opportunities early.

In a historic milestone for global health equity, the Global Fund recently signed an access agreement with Gilead Sciences to procure lenacapavir for low- and middle-income countries (LMICs). This is the first time an HIV prevention product will be introduced in LMICs simultaneously with high-income countries, underscoring our commitment to ensuring rapid, affordable, and equitable access to life-saving innovations.

## Replenishment in an era of uncertainty

The upcoming 2025 Global Fund Replenishment – co-hosted by South Africa and the United Kingdom – arrives at a moment of both risk and opportunity. The Replenishment must serve not only as a funding mechanism, but also as a recommitment to the values of global solidarity and shared responsibility.

This Replenishment is about reaffirming the principles that have made the global HIV response one of the most effective mobilizations in public health history. Donors must step up not because it's easy, but because it's right – and necessary.

## Discussion

The fight against HIV and AIDS is a powerful testament to human ingenuity, resilience and collaboration. But it remains unfinished.

To sustain momentum, we must go beyond financing. Strong national, inclusive governance and resilient health systems capable of addressing comorbidities and delivering integrated care are essential. While the Global Fund has provided critical support, long-term success increasingly hinges on greater domestic investment and reduced reliance on external aid (3, 4). Linking HIV services with maternal health, tuberculosis, and non-communicable disease management is especially in resource-limited settings (5, 6).

At the same time, the geographic contours of the epidemic are shifting. The rise in HIV incidence in Eastern Europe, Central Asia, and the Middle East demands locally tailored strategies that confront structural drivers like criminalization, discrimination and marginalization.

While biomedical innovation is transforming HIV prevention and care, no single tool will end the disease as a public health threat. Behavioral interventions, stigma reduction and legal reform remain indispensable. Strengthening pharmacovigilance systems to monitor drug resistance and emerging threats is equally critical (7, 8).

## Conclusion

Ending AIDS is possible – but only if we remain steadfast. The Global Fund's Eighth Replenishment this year is not just an opportunity to raise resources: It is a test of our shared commitment to finishing what we started.

The reductions in donor financing for health, which gathered pace in 2024 and sharply accelerated in 2025, underscore the urgent need for countries to accelerate the transition to nationally led and nationally financed health systems – ones no longer reliant on external support. Yet this is a pathway, not a switch. Too much abrupt transition will derail progress, leave the vulnerable behind, and cost millions of lives. Moreover, countries vary widely in their readiness to undertake this transition.

The Global Fund acts as an enabler on this pathway – encouraging, supporting, and incentivizing countries to take leadership, while ultimately stepping back as they assume full ownership. Accelerating this journey to self-reliance is critical to sustaining and building upon the hard-won gains in the fight against HIV and AIDS.

If we act decisively, invest equitably and uphold the inclusive, rights-based approach that has defined the global HIV response, we can consign AIDS to history. But we must not falter now. The last mile is always the hardest – but the most important.
